# Mediating effects of patient safety perception and willingness to participate in patient safety on the relationship between health literacy and patient participation behavior among inpatients

**DOI:** 10.3389/fpubh.2024.1349891

**Published:** 2024-02-13

**Authors:** Mi Hwa Won, Sun-Hwa Shin

**Affiliations:** ^1^Department of Nursing, Wonkwang University, Iksan, Republic of Korea; ^2^College of Nursing, Sahmyook University, Seoul, Republic of Korea

**Keywords:** inpatients, patient safety, perception, patient participation, health literacy

## Abstract

**Introduction:**

In recent years, patient safety activities have shifted from being centered on healthcare providers to involving patients themselves. Health literacy of inpatients has a direct impact on patient participation behavior. Patient safety perception was also associated with willingness to participate in patient safety and patient participation behavior. Therefore, this study aimed to investigate the mediating effects of patient safety perception and willingness to participate in patient safety on the relationship between health literacy and patient participation behavior among inpatients.

**Methods:**

This cross-sectional study was conducted to confirm the relationship between study variables. A total of 262 inpatients were recruited from patients admitted to the ward of a tertiary general hospital between October and November 2023. Participants were invited to complete self-reported questionnaires that measured health literacy, patient safety perception, willingness to participate in patient safety, patient participation behavior, and demographic information. Data were analyzed using a dual mediation model applying the PROCESS macro (Model 6) with 95% bias-corrected bootstrap confidence intervals.

**Results:**

This study analyzed the direct effects of health literacy on patient safety perceptions and patient participation behavior. Health literacy indirectly affected patient participation behavior through patient safety perceptions and willingness to participate in patient safety. Regarding the relationship between health literacy and patient participation behavior, patient safety perceptions and willingness to participate in patient safety showed a significant dual mediating effect.

**Conclusions:**

This study identified the factors that promote patient participation behavior among inpatients. The mediating effect of patient safety perception on the relationship between health literacy and patient participation behavior was found to be strong. Building health literacy among inpatients ensures patient safety by increasing patient safety perceptions.

## 1 Introduction

Patient safety refers to minimizing the risk of harm or error to patients during the delivery of healthcare services (1). One in 10 hospitalized patients suffers harm while receiving medical services, and it is reported that more than 3 million people experience fatal harm due to unsafe medical services every year (2). More than 50% of the harm is reportedly preventable (3, 4), and medication errors, unsafe surgical procedures, healthcare-associated infections, diagnostic errors, falls, pressure ulcers, and patient identification errors can be prevented through patient safety activities (1). Much of the research on patient safety has focused on preventing errors and mistakes in the delivery of healthcare, with particular emphasis on healthcare providers. Recently, however, it has been suggested that actively involving the parties directly affected by patient safety incidents in patient safety activities is the best way to prevent medical errors (5). In other words, patient safety has shifted from a healthcare provider-centered activity to a patient-directed one (6, 7). So active patient participation behavior is critical to reducing the risk of medical errors.

Health literacy is the ability to use information to make decisions and effectively care for one's health, and a patient's health literacy is an important determinant of health (8, 9). Patients with higher health literacy are more likely to understand healthcare providers' explanations and engage in disease-related preventive behaviors (10, 11). In contrast, patients with low health literacy did not adequately manage their health, such as failing to understand treatment prescriptions, not asking questions about medical issues, and not attempting to seek new health information (10). In addition, patients with low health literacy may have problems with communication critical to patient safety, such as taking medications at doses different from those prescribed (12). Inpatient health literacy directly influences patient safety engagement perceptions and acts as a mediator in the relationship between the patient safety environment and patient safety engagement perceptions (13). In this way, health literacy, a patient's personal characteristic, is a major factor affecting patient participation behavior.

Patient safety perception is the level at which patients perceive that their safety is not threatened in the medical field (14). Therefore, patient safety perception refers to the degree to which patients are aware of situations that pose a risk to their safety during the process of receiving medical services and how to prevent them (15, 16). Previous studies have shown that a higher patient safety perception among inpatients is associated with a higher willingness to engage in patient safety (15) and that patient safety perception is positively correlated with the performance of patient safety activities (17). To increase inpatient engagement in patient safety, it is necessary to enhance the perception that patients play a key role in ensuring their safety (17, 18).

Willingness to participate in patient safety is a patient's willingness to take a more active role in patient safety activities, which means taking an interest in their care and taking the initiative to participate in the care process (19). This can improve patient safety through the identification of possible side effects and increased effectiveness of treatment by monitoring patient progress (20). Factors that affect willingness to participate in patient safety include patient-related factors (age, gender, and health status), disease-related factors (underlying disease, severity, and patient safety accident experience), medical staff-related factors (knowledge and beliefs), and medical institution-related factors (type of medical institution and patient safety work type) (19). Inpatients' patient safety perception was a factor that affects willingness to participate in patient safety (15).

Patient participation behavior refers to a patient's participation in the decision-making processes related to health issues. Furthermore, it is the act of taking the initiative to engage patients in various aspects of healthcare, such as self-medication, self-monitoring, patient education, goal setting, and physical care (21). It has been reported that inpatients have limited participation in the care process and low levels of perception of what patient safety activities (7). In addition, the higher the patient safety perception of inpatients, the higher the patient participation behavior level (16). Factors that hinder patient participation include acceptance of new patient roles, low levels of health literacy, lack of confidence in competency, and the presence of diseases and comorbidities (22). In the past, it was assumed that deferring to medical decision-making was the best option for treatment. It is now recommended to listen to patients and involve them in the decision-making process by involving them in all aspects of their care (19). Therefore, interaction between medical staff and patients is an important factor in promoting patient safety (19, 21), and patients themselves need to practice taking an interest in their health and actively participating in decisions related to their care (11).

Previous studies have shown that the health literacy of inpatients has a direct impact on patient participation behavior (13, 23). Additionally, patient safety perception is related to willingness to participate in patient safety (15), and patient safety perception is related to patient participation behavior (16, 17). Health literacy is an individual's ability to make decisions about health, and it was judged to be related to patient safety perception and willingness to participate in patient safety; therefore, the following hypothesis was set: The health literacy of inpatients influences patient participation through patient safety perception and willingness to participate in patient safety.

The specific purposes of this study were as follows: First, the correlations between health literacy, patient safety perceptions, willingness to participate in patient safety, and patient participation behavior will be identified. Second, the mediating path that influences the patient participation behavior will be identified.

## 2 Methods

### 2.1 Study design and participants

This descriptive study examined the correlations between health literacy, patient safety perception, willingness to participate in patient safety, and patient participation behavior among inpatients. Participants were patients admitted to the general ward of a tertiary general hospital with more than 700 beds in South Korea. The criteria for selecting participants were: those who had previously been hospitalized, adults over 20 years of age, those with a clear state of consciousness and the ability to communicate, and those who understood the purpose of the study and agreed to voluntarily participate. Exclusion criteria were those who had never been hospitalized; those with a history of stroke or vascular dementia, memory impairment, or mental illness; those taking psychiatric drugs such as antidepressants; and those diagnosed with chronic renal failure or terminal cancer. From the data of 295 people who met the selection criteria, 33 who had no previous hospitalization were removed, and the final data of 262 people were used for the analysis.

A *post-hoc* test for multiple regression analysis was performed using G^*^Power (version 3.1.9.2) to examine the adequacy of the sample size. Since there were no prior studies suggesting the effect size, a medium effect size (f^2^ = 0.15) was set, the significance level (α) was 0.05, and the power of the data was 98.9% when analyzing data from 262 people based on 16 predictive factors. The sample size for the analysis was judged to be appropriate.

### 2.2 Measures

#### 2.2.1 Health literacy

Health literacy was measured using the Korean version of the Brief Health Literacy Screener developed by Chew et al. (24) and the Brief Health Literacy Screener (25) to assess individuals' ability to access and utilize health-related information. The instrument has three questions, each rated on a 5-point Likert scale from 0 to 4, with scores ranging from 0 to 12. Higher score indicates a higher level of health literacy. In the study by Son and Song (25), the internal consistency coefficient (Cronbach's α) of the instrument was 0.82, while in the present study it was 0.75.

#### 2.2.2 Patient safety perception

The patient safety perception of Korean inpatients was measured using the Patient Safety Perception Scale developed by Kim et al. (14). The instrument was developed based on previous research and qualitative interviews, and its content, criteria and construct validity were verified. The instrument has 24 questions, each rated on a 5-point Likert scale from 1 to 5, with scores ranging from 24 to 120. A higher score indicates a higher level of patient safety perception. In the study by Kim et al. (14), the internal consistency coefficient (Cronbach's α) of the instrument was 0.93, while in the present study it was 0.95.

#### 2.2.3 Willingness to participate in patient safety

Patients' willingness to participate in patient safety was measured using the Willingness toward Participation in Patient Safety instrument developed by Lee (26). The instrument was developed based on a literature review of 20 guidelines for preventing medical errors published by the Agency for Healthcare Research and Quality (AHRQ), and its content validity was verified by experts. The instrument has 18 questions, each rated on a 4-point Likert scale from 1 to 4, with scores ranging from 18 to 72. A higher score indicates a higher level of willingness to participate in patient safety. In the study by Lee (26), the internal consistency coefficient (Cronbach's α) of the instrument was 0.88, while in the present study it was 0.94.

#### 2.2.4 Patient participation behavior

Patient participation behavior was measured using the Patient Participation Scale developed by Song and Kim (27) for outpatients and inpatients. The instrument was developed based on prior research and was validated for content validity, construct validity, and reliability. The instrument has 21 questions, each rated on a 5-point Likert scale from 1 to 5, with scores ranging from 21 to 105. A higher score indicates a higher level of patient participation. In the study by Song and Kim (27), the internal consistency coefficient (Cronbach's α) of the instrument was 0.92, while in the present study it was 0.93.

### 2.3 Data collection and ethical considerations

This study was approved by the Institutional Review Board of the investigator's university (IRB no: WKIRB-202310-SB-079). The data were collected from October to November 2023, and a face-to-face survey was conducted among inpatients in the ward of a tertiary general hospital. After obtaining permission from the relevant institution, study participants were recruited through postings. An informed consent document was presented to the participants to explain the purpose, content, and procedures of the study. In addition, it was explained in advance that they could freely choose to stop answering the survey or withdraw their consent, and that there would be no disadvantage. After receiving an explanation of the study, participants signed a consent form for voluntary participation and completed the survey. Details on how to ensure the confidentiality of the collected data were provided. Those who completed the survey were compensated for their participation.

### 2.4 Data analysis

SPSS 25.0 (IBM Institute, NY, USA) was used to analyze the data. The general characteristics of the participants were analyzed using descriptive statistics of frequency and percentage, mean, and standard deviation. Differences in the study variables (health literacy, patient safety perception, willingness to participate in patient safety, and participation behavior) according to general characteristics were analyzed using an independent *t*-test, one-way ANOVA, and *post-hoc* Scheffé's test. The relationships between the study variables were analyzed using Pearson's correlation. The reliability of the instrument was checked with the Cronbach's α value. To test the significance of the indirect effect, the PROCESS procedure for SPSS Version 4.1 (Model 6) was used. The 95% confidence intervals (CIs) were calculated using bootstrapping and the mediating effect was considered significant if the lower and upper bounds of the 95% CIs did not include zero. The significance of the size of the mediation effect and the differences in the path of the mediation effect were verified. All analyses had a statistical significance level of 0.05 or less.

## 3 Results

### 3.1 Differences in patient participation behavior based on general characteristics

The general characteristics of the study participants are presented in Table 1. There were 149 men (56.9 %) and 113 women (43.1 %). The average age was 62.65 years (±18.03), with 140 (53.4%) aged 65 or older. The most common level of education was middle school or lower (111, 42.4%). In total, 156 (59.5%) patients were unemployed. Regarding residence type, 113 (43.1%) lived with several people as a family or group, and 100 (38.2%) lived alone with their spouse. Most patients were in the internal medicine department (132 patients, 50.4%) and patients with surgical experience accounted for more than half (185 patients, 70.6%). There were 139 (53.1%) patients who received patient safety education upon admission and 123 (46.9%) who did not. A total of 163 patients (62.2%) had underlying diseases (cardiovascular system, diabetes, stroke, etc.) and 155 patients (59.2%) were taking medications (antihypertensive drugs, oral hypoglycemic agents, hyperlipidemic drugs, diuretics, etc.).

**Table 1:** Demographic characteristics of participant (*N* = 262).

**Characteristics**	**Categories**	***n* (%)**
Gender	Men	149 (56.9)
	Women	113 (43.1)
Age (year)	≦64	122 (46.6)
	≧65	140 (53.4)
Education level	Middle school	111 (42.4)
	High school	87 (33.2)
College or higher	64 (24.4)
Occupation	Don't have	156 (59.5)
	Have	106 (40.5)
Residential status	Living alone	49 (18.7)
	Living with a spouse	100 (38.2)
	Live together	113 (43.1)
Medical department	Internal medicine	132 (50.4)
	Surgical department	86 (32.8)
	Others (urology, plastic, etc.)	44 (16.8)
Surgical experience	No	77 (29.4)
	Yes	185 (70.6)
Patient safety training	No	123 (46.9)
	Yes	139 (53.1)
Illness history	No	99 (37.8)
	Yes	163 (62.2)
Medication	No	107 (40.8)
	Yes	155 (59.2)

Descriptive statistics in the study variables according to the general characteristics are shown in Table 2. Health literacy differed significantly by age (*t* = 5.06, *p* < 0.001), education level (*F* = 25.55, *p* = 0.001), occupation (*t* =−2.72, *p* = 0.007), underlying disease (*t* = 3.69, *p* < 0.001), and use of oral medication (*t* = 3.29, *p* = 0.001). *Post-hoc* tests for education level showed that college and high school graduates had higher health literacy than those with a high school diploma or lower. Patient safety perception was significantly different by patient safety education (*t* =−2.75, *p* = 0.006). The willingness to participate in patient safety differed significantly by gender (*t* =−2.29, *p* = 0.023) and patient safety education (*t* =−2.50, *p* = 0.013). Patient participation behavior was significantly different based on patient safety education (*t* =−2.40, *p* = 0.017).

**Table 2:** Descriptive statistics in health literacy, patient safety perception, willingness to participation in patient safety, and patient participation behavior by demographic characteristics (*N* = 262).

**Characteristics**	**Categories**	**Health literacy**		**Patient safety perception**		**Willingness to participate in patient safety**		**Patient participation behavior**	
		**M** ±**SD**	**t/F (** ***p*** **)**	**M** ±**SD**	**t/F (** ***p*** **)**	**M** ±**SD**	**t/F (** ***p*** **)**	**M** ±**SD**	**t/F (** ***p*** **)**
Gender	Men	2.15 ± 0.81	1.27 (0.207)	4.18 ± 0.63	−0.84 (0.404)	3.27 ± 0.50	−2.29 (0.023)	3.89 ± 0.62	−1.60 (0.111)
	Women	2.01 ± 1.01		4.24 ± 0.57		3.41 ± 0.46		4.01 ± 0.57	
Age (year)	≦64	2.37 ± 0.71	5.06 (< 0.001)	4.22 ± 0.55	0.43 (0.671)	3.36 ± 0.44	0.94 (0.348)	3.93 ± 0.55	−0.16 (0.874)
	≧65	1.84 ± 0.97		4.19 ± 0.64		3.31 ± 0.53		3.95 ± 0.64	
Education level	Middle school^a^	1.67 ± 0.94	25.55 (< 0.001) a < b,c	4.17 ± 0.66	0.56 (0.573)	3.32 ± 0.54	0.17 (0.843)	3.99 ± 0.64	0.58 (0.558)
	High school^b^	2.47 ± 0.76		4.26 ± 0.56		3.33 ± 0.43		3.90 ± 0.58	
	College or higher^c^	2.29 ± 0.67		4.19 ± 0.56		3.36 ± 0.47		3.91 ± 0.55	
Occupation	Don't have	1.96 ± 0.98	−2.72 (0.007)	4.18 ± 0.63	−0.91 (0.364)	4.18 ± 0.63	−0.35 (0.728)	4.18 ± 0.63	0.17 (0.865)
	Have	2.27 ± 0.72		4.25 ± 0.55		4.25 ± 0.55		4.25 ± 0.55	
Residential status	Living alone	1.87 ± 1.07	2.29 (0.104)	4.04 ± 0.80	2.90 (0.057)	3.38 ± 0.58	0.32 (0.723)	3.84 ± 0.73	1.60 (0.204)
	Living with a spouse	2.20 ± 0.77		4.29 ± 0.51		3.31 ± 0.47		4.02 ± 0.54	
	Live together	2.08 ± 0.91		4.20 ± 0.57		3.34 ± 0.46		3.91 ± 0.58	
Medical department	Internal medicine	1.95 ± 0.88	3.03 (0.051)	4.19 ± 0.66	0.09 (0.917)	3.31 ± 0.52	0.35 (0.703)	3.92 ± 0.67	0.47 (0.624)
	Surgical department	2.22 ± 0.91		4.23 ± 0.53		3.34 ± 0.46		3.94 ± 0.50	
	Others (urology, plastic, etc.)	2.23 ± 0.87		4.19 ± 0.58		3.38 ± 0.44		4.02 ± 0.57	
Surgical experience	No	2.10 ± 0.75	0.40 (0.919)	4.26 ± 0.65	0.88 (0.378)	3.34 ± 0.53	0.11 (0.915)	3.93 ± 0.64	−0.22 (0.823)
	Yes	2.08 ± 0.95		4.18 ± 0.58		3.33 ± 0.47		3.95 ± 0.58	
Patient safety training	No	2.04 ± 0.93	−0.78 (0.438)	4.10 ± 0.64	−2.75 (0.006)	3.25 ± 0.50	−2.50 (0.013)	3.85 ± 0.59	−2.40 (0.017)
	Yes	2.13 ± 0.87		4.30 ± 0.55		3.40 ± 0.46		4.02 ± 0.60	
Illness history	No	2.34 ± 0.74	3.69 (< 0.001)	4.19 ± 0.59	−0.25 (0.805)	3.37 ± 0.48	0.85 (0.398)	3.92 ± 0.56	−0.50 (0.617)
	Yes	1.93 ± 0.95		4.21 ± 0.61		3.31 ± 0.49		3.96 ± 0.62	
Medication	No	2.30 ± 0.76	3.29 (0.001)	4.20 ± 0.58	−0.02 (0.983)	3.37 ± 0.49	1.01 (0.314)	3.91 ± 0.59	−0.75 (0.457)
	Yes	1.94 ± 0.96		4.21 ± 0.62		3.31 ± 0.49		3.96 ± 0.60	

^a, b, c^Alphabets refer to *post-hoc* test results using Scheffé's method.

### 3.2 Description and correlations of study variables

The mean scores of the study variables are presented in Table 3. Health literacy averaged 2.09 (±0.90), patient safety perception 4.20 (±0.60), willingness to participate in patient safety 3.33 (±0.49), and patient participation behavior 3.94 (±0.60). As a result of examining the skewness and kurtosis of the research variables, the skewness was within ±2 and the kurtosis was within ±4, forming a normal distribution.

**Table 3:** Descriptive statistics and correlation of health literacy, patient safety perception, willingness to participate in patient safety, and patient participation behavior (*N* = 262).

**Variables**	**PSP**	**WP**	**PPB**	**Mean ±SD**	**Skewness**	**Kurtosis**
	**r (** ***p*** **)**					
HL	0.14 (0.026)	0.08 (0.179)	0.13 (0.034)	2.09 ± 0.90	−0.18	0.08
PSP		0.62 (< 0.001)	0.70 (< 0.001)	4.20 ± 0.60	−0.97	2.62
WP			0.70 (< 0.001)	3.33 ± 0.49	−0.65	1.23
PPB				3.94 ± 0.60	−0.53	1.47

HL, Health literacy; PSP, Patient safety perception; WP, Willingness to participate in patient safety; PPB, Patient participation behavior.

Table 3 presents the results of correlation analyses of the study variables. Health literacy was significantly positively correlated with patient safety perception (*r* = 0.14, *p* = 0.026) and patient participation behavior (*r* = 0.13, *p* = 0.034). However, health literacy did not correlate with willingness to participate in patient safety in the study (*r* = 0.08, *p* = 0.179). Patient safety perception was significantly positively correlated with willingness to participate in patient safety (*r* = 0.62, *p* < 0.001) and patient participation behavior (*r* = 0.70, *p* < 0.001). Willingness to participate in patient safety was significantly positively correlated with patient participation behavior (*r* = 0.70, *p* < 0.001).

### 3.3 Significance of the mediating effect of patient safety perception and willingness to participate in patient safety and differences in the mediating effect by pathway

The results of the dual mediation effect of patient safety perception and willingness to participate in patient safety in the relationship between health literacy and patient participation behavior are shown in Table 4. A mediation analysis was performed after including gender, age, and patient safety education as control variables, which showed a significant difference in patient participation behavior among the general characteristics. In Model 1, the independent variable, health literacy, had a significant positive effect (β = 0.15, *p* = 0.020) on the primary parameter, patient safety perception (R^2^ = 0.051, *F* = 3.43, *p* = 0.001). In Model 2, health literacy had no significant effect on the secondary parameter, willingness to participate in patient safety (β =−0.01, *p* = 0.912), while the primary parameter, patient safety perception, had a significant static effect (β = 61, *p* < 0.001) on willingness to participate in patient safety (R^2^ = 0.398, *F* = 33.89, *p* < 0.001). In Model 3, where two parameters were entered simultaneously, the first parameter, patient safety perception, had a significant positive effect (β = 0.42, *p* < 0.001) and the second parameter, willingness to participate in patient safety, had a significant positive effect (β = 0.44, *p* < 0.001) on patient participation behavior. However, health literacy did not have a significant effect on patient participation behavior (β = 0.06, *p* = 0.130). The final model had an explanatory power of 61.6% (R^2^ = 0.616, *F* = 68.02; *p* < 0.001).

**Table 4:** Results of mediating effect analysis (*N* = 262).

**Model**	**DV**	**IV**	**B**	**SE**	**β**	**t**	***p***	**Adj. R^2^**	**F (*p*)**
1	PSP	HL	0.10	0.04	0.15	2.33	0.020	0.051	3.43 (0.009)
2	WP	HL	−0.01	0.03	−0.01	−0.11	0.912	0.398	33.89 (< 0.001)
		PSP	0.49	0.04	0.61	12.17	< 0.001		
3	PPB	HL	0.04	0.03	0.06	1.52	0.130	0.616	68.02 (< 0.001)
		PSP	0.42	0.05	0.42	8.34	< 0.001		
		WP	0.54	0.06	0.44	8.76	< 0.001		

DV, Dependent variable; IV, Independent variable; Adjusted for gender, age, and patient safety education; HL, Health literacy; PSP, Patient safety perception; WP, Willingness to participate in patient safety; PPB, Patient participation behavior.

The significance of the mediating effect (indirect effect) of patient safety perception and willingness to participate in patient safety in the relationship between health literacy and patient participation behavior was analyzed and is presented in Table 5. The mediating effect of health literacy on patient participation behavior through patient safety perception (Indirect 1) was statistically significant, as the lower and upper 95% confidence intervals did not include zero [*B* = 0.04, boot 95% CI (0.01, 0.09)]. However, the mediating effect of health literacy on patient participation behavior through willingness to participate in patient safety (Indirect 2) was not statistically significant [*B* = −0.01, boot 95% CI (−0.03, 0.03)]. Finally, the dual mediation of patient safety perception and willingness to participate in patient safety (Indirect 3) was statistically significant [*B* = 0.03, Boot 95% CI (0.01, 0.06)]. As a result of checking for a difference in the mediation effect (indirect effect) by path, no significant difference was found among the three mediation paths.

**Table 5:** Significance test of mediating effects of patient safety perception and willingness to participate in patient safety (*N* = 262).

**Model**	**Variables**	**Direct effect**				**Indirect effect**			
		**Effect**	**Boot SE**	**95% CI**		**Effect**	**Boot SE**	**95% CI**	
				**Boot LLCI**	**Boot ULCI**			**Boot LLCI**	**Boot ULCI**
Direct	HL → PPB	0.11	0.43	0.03	0.20				
Indirect 1	HL → PSP → PPB					0.04	0.02	0.01	0.09
Indirect 2	HL → WP → PPB					−0.01	0.02	−0.03	0.03
Indirect 3	HL → PSP → WP → PPB					0.03	0.02	0.01	0.06
Differences (ΔB)	Indirect 1 - Indirect 2					0.04	0.02	−0.01	0.09
	Indirect 1 - Indirect 3					0.02	0.01	−0.01	0.04
	Indirect 2 - Indirect 3					−0.03	0.02	−0.07	0.01

CI, confidence interval; LLCI, lower-level confidence interval; ULCI, upper-level confidence interval. HL, health literacy; PSP, patient safety perception; WP, willingness to participate in patient safety; PPB, patient participation behavior.

**Figure 1:**
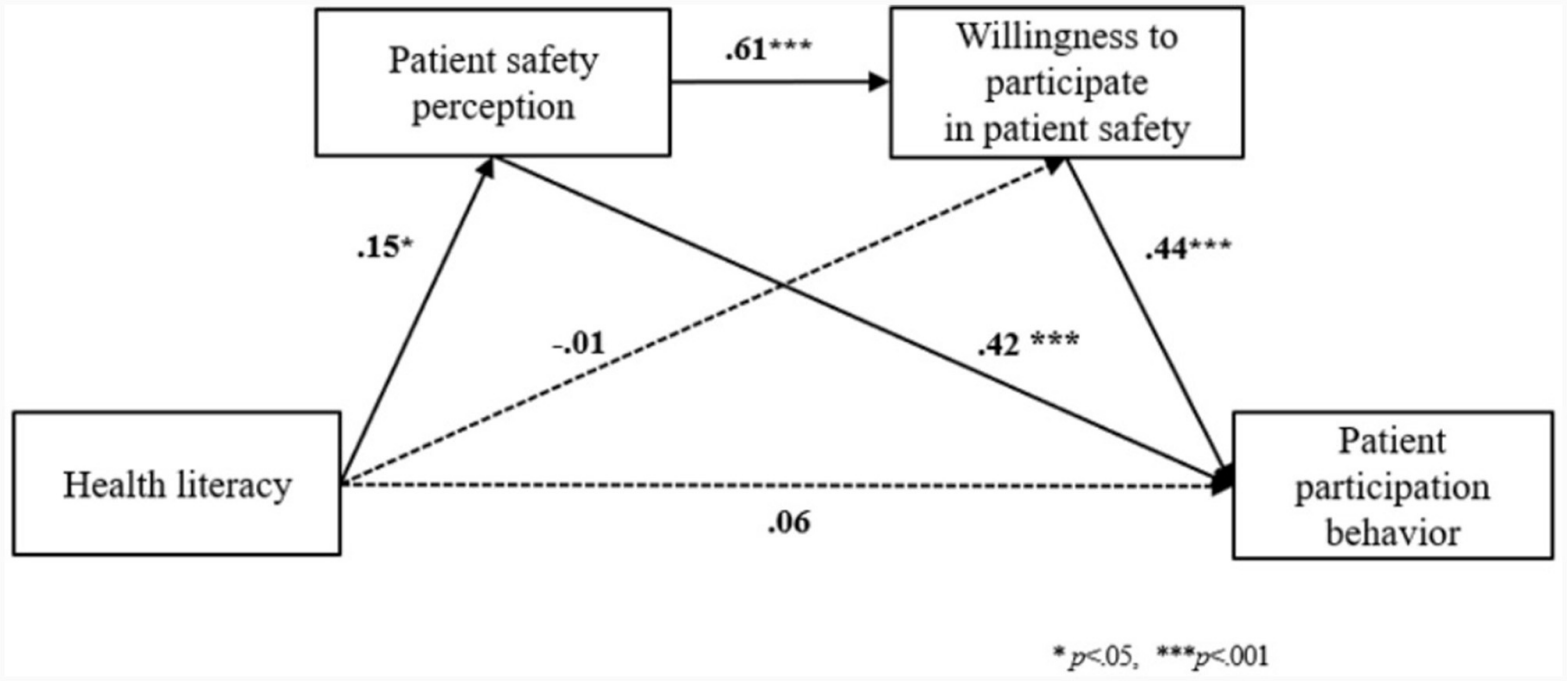
The dual mediation effect of patient safety perception and willingness to participate in patient safety in the relationship between health literacy and patient participation behavior. The solid and dashed line indicated significant and non-significant path coefficients.

Two pathways were identified in this study. We found that the mediating effect of health literacy on patient participation behavior by increasing patient safety perception (*B* = 0.04) and the mediating effect of health literacy on patient participation behavior by increasing patient safety perception and willingness to participate in patient safety (*B* = 0.03) were significant. These findings suggest a slightly stronger mediating effect of patient safety perception on the relationship between health literacy and patient participation behavior (Figure 1).

## 4 Discussion

This study conducted a mediating effects analysis to examine the correlations among health literacy, patient safety perception, willingness to participate in patient safety, and patient participation behavior among inpatients.

The health literacy of inpatients had a significant direct effect on their participation behavior. This finding aligns with previous research that validated the role of health literacy in promoting patient participation behavior among inpatients (13, 22). Variables showing significant differences in the health literacy of the inpatients included age and educational level. Similar to results from studies targeting inpatients, lower age and higher educational levels were associated with higher health literacy in this study (13, 28, 29). Furthermore, we found that patients with jobs demonstrated higher health literacy than those without. While a study focusing on stroke patients did not reveal differences in health literacy based on occupational status, it indicated lower health literacy in patients with lower monthly income (29), suggesting that economic factors, such as occupation and income, may influence health literacy. In addition, this study observed that inpatients without underlying diseases and those not taking oral medications exhibited higher levels of health literacy than those with such conditions. Despite the expectation that patients with underlying diseases or on medication should excel in self-management, the lower health literacy observed in these groups emphasizes the need to highlight health information related to diagnosed diseases and prescribed medications, along with conducting periodic education. Glick et al. (23) advocated the development of interventions based on health literacy, integrating strategies for patients, families, and the healthcare system to improve treatment outcomes and enhance patient- and family-centered care. Future research should explore various factors influencing health literacy among inpatients diagnosed with diseases to formulate strategies for tailored education.

The health literacy of inpatients exerted a significant direct impact on patient participation behavior through the dual mediation of patient safety perceptions and willingness to participate in patient safety. Health literacy did not directly influence willingness to participate in patient safety; instead, it affected willingness through patient safety perception. The confirmation that personal factors of health literacy play a crucial role in determining patient safety perceptions among inpatients is meaningful. Jang and Park (13) reported in their study that health literacy among inpatients acts as a mediator between the patient safety environment and participation, suggesting that patient safety participation may vary depending on health literacy. A qualitative study targeting inpatients demonstrated positive outcomes, indicating that active participation in the decision-making process for treatment and sharing health information leads to a desire for active involvement not only in personal treatment, but also in inpatient safety activities (30). Thus, beyond mere knowledge, health literacy represents the ability of patients to process the information and services necessary to make informed decisions about their healthcare (31), contributing to an increase in patient safety perception among inpatients. Moreover, an increase in patient safety perception, which can play a central role in ensuring patient safety, is expected to enhance the willingness and behavior of inpatients to participate in patient safety efforts (18).

Patient safety perception among inpatients have emerged as a powerful factor that contributes to increased patient willingness and participation behavior. Previous research has demonstrated a positive, static correlation between patient safety perception and the performance of patient safety activities (17). Additionally, patient safety perception was found to enhance willingness to participate in patient safety (15). Patient safety perception has been identified as a contributing factor to increased patient participation behavior in previous studies (16), which aligns with the results of the present study. To enhance patient safety within healthcare institutions, strategies involving the creation of a safe healthcare environment and analysis of the causes of patient safety incidents are imperative (32). The current reporting and learning system for patient safety incidents in South Korea primarily involves reporting by designated patient safety personnel, with patient and caregiver reports constituting a minimal portion (33). Despite patients experiencing temporary or long-term harm and side effects from medical care, voluntary reporting remains relatively low. Although campaigns and activities related to patient safety are conducted within healthcare institutions, there is currently a lack of education and promotion targeting patients and non-medical individuals. This deficiency, coupled with a societal atmosphere in which patients are not inclined to voluntarily report even minor medical errors, poses a challenge. To actively promote inpatient participation, a strategic approach is required to elevate patient safety perceptions by promoting various medical and patient safety activities within healthcare institutions.

Patient safety education has acted as a factor elevating patient participation behavior. Previous studies conducted on inpatients indicated that after implementing patient safety education, there was an increase in patient safety perceptions (34, 35) and behaviors (36). An increase in patient safety knowledge through education has been identified as a factor that enhances health literacy and promotes patient participation behavior (22). However, there is limited research on the effectiveness of patient safety education for inpatients and their caregivers. It is crucial for inpatients to possess an active willingness to participate in ensuring their own safety; therefore, patient safety education targeting inpatients should be prioritized (34). Inpatients often find themselves inundated with various educational materials during their hospital stay, leading to a lack of interest in patient safety education (37). In addition, they tended to focus only on disease education to achieve their hospitalization goals (38). Given the frequent transitions between hospitalization and discharge, education provided by the assigned nurse or patient safety specialist may lack continuity. Therefore, it is necessary to standardize easily understandable educational materials for inpatients and caregivers (37). Furthermore, the development of tailored patient safety education is warranted, considering the characteristics and severity of the patient's illness and differentiating the timing of applying education.

Suggestions based on the limitations of this study were as follows. As the research was conducted with inpatients from a single hospital, caution is needed when generalizing the study results. Therefore, future research should expand the sample to be more diverse, including not only tertiary comprehensive hospitals but also smaller hospitals, to ensure the validity of the research findings. The characteristics of inpatients admitted to tertiary hospitals can vary. This diversity may lead to differences in health information and patient safety-related content, based on factors such as the severity of the patient's condition, pain levels, and surgical history, thereby limiting our understanding of the impact on patient participation behavior. Therefore, in future research, it is essential to acknowledge these limitations and consider the various factors that may influence patient participation behavior to ensure the generalizability of the research results. Finally, considering the relationship between health literacy, patient safety perceptions, and willingness to participate in patient safety, it is recommended that a tailored patient safety education program for inpatients be developed to enhance patient participation behavior. Future research should aim to validate the effectiveness of these programs.

## 5 Conclusion

This study identified the factors that promote patient participation behavior among inpatients. The results revealed that health literacy, patient safety perception, and willingness to participate in patient safety directly influenced patient participation behavior. Additionally, by verifying the dual mediating effects of patient safety perception and patient willingness in the relationship between health literacy and patient participation behavior, this study provides a foundation for understanding the structure of these concepts. There were no differences in the paths of the three mediating effects, and the mediating effect of patient safety perception played a slightly stronger role in the relationship between health literacy and patient participation behavior. Based on the findings of this study, it is necessary to implement effective patient safety education and promote programs to enhance patients' active participation behaviors during hospitalization. This includes establishing high health literacy regarding illnesses and increasing patient safety perception and willingness to participate in patient safety.

## Data availability statement

The original contributions presented in the study are included in the article/supplementary material, further inquiries can be directed to the corresponding author.

## Ethics statement

The studies involving humans were approved by the Institutional Review Board of Wonkwang University Hospital. The studies were conducted in accordance with the local legislation and institutional requirements. Written informed consent for participation in this study was provided by the participants' legal guardians/next of kin.

## Author contributions

MW: Data curation, Formal analysis, Methodology, Project administration, Supervision, Validation, Writing—original draft, Writing—review & editing, Investigation. S-HS: Formal analysis, Funding acquisition, Methodology, Resources, Software, Writing—original draft, Writing—review & editing, Conceptualization.
